# Current status of global dairy goat production: an overview

**DOI:** 10.5713/ajas.19.0253

**Published:** 2019-07-01

**Authors:** Beth A. Miller, Christopher D. Lu

**Affiliations:** 1 Department of Natural and Physical Sciences, University of Arkansas - PTC, North Little Rock, AR 72118, USA; 2 College of Agriculture, Forestry and Natural Resource Management, University of Hawaii, Hilo, HI 96720, USA

**Keywords:** Asia, Dairy Goats, Europe, Goat Cheese, Goat Milk, United States

## Abstract

The global goat population continues to grow and is now over one billion. The number of goats raised primarily for milk production is also growing, due to expanding demand. Most of the world dairy goat production and consumption is in Asia, but a global view of the dairy goat sector reveals important lessons about building successful modern dairy goat industries. The most organized market for goat milk is found in Europe, especially in France. The European goat sector is specialized for milk production, mostly for industrial cheesemaking, while also supporting traditional on-farm manufacturing. Government involvement is significant in sanitary regulation, research, extension, support for local producer organizations, and markets, and ensures safety and quality. Nonetheless, producers are still vulnerable to market fluctuations. New dairy goat industries are developing in countries without a long goat milk tradition, such as China, the United States, and New Zealand, due to rising consumer demand, strong prices, and climate change. The mix of policies, management and markets varies widely, but regardless of the country, the dairy goat sector thrives when producers have access to markets, and the tools and skills to sustainably manage their livestock and natural resources. These are most readily achieved through strong and inclusive producer organizations, access to technical services, and policies that enable the poor and marginalized groups to benefit from increasing demand.

## INTRODUCTION

The global dairy goat industry is expanding rapidly. In addition to wholesome and nutritious milk-based products, dairy goats provide sustainable livelihoods, especially in limited resource areas, and enable smallholders to accumulate assets. Well-managed goats can also benefit the environment through weed control, fire prevention, the maintenance of biodiversity, and mitigation of some effects of climate change. This is largely attributed to their diet selection and eating behaviors [1,2]. In addition to milk, dairy goats provide other benefits to owners, including skins for leather, kids for meat, and manure for fertilizer, and can add revenue streams when they graze under trees on oil palm plantations.

The International Goat Association (IGA) has been the global advocate for the goat sector since 1982, and has contributed to the expansion of goat knowledge and practice through conferences, the academic journal *Small Ruminant Research*, and social media. Researchers, producers and policy makers working together and sharing information and experiences will enable the sector to reach its full potential. With its mission to promote goat research and development for the benefit of humankind, to alleviate poverty, to promote prosperity, and to improve the quality of life, IGA is the only organization that supports the goat sector in every corner of the world. It is a global network of people and organizations that links research, production, processing, and marketing; shares information, experience, and best practices; advocates socially just, environmentally sound, and economically viable goat production; and promotes international, regional, and local activities with global and diverse perspectives [3].

The global goat population has been rising dramatically since the 1960s, due to changing incomes and food preferences in human populations, and climate change limiting areas for raising cattle. In 2013, the global goat herd was over one billion head, a rise of over 34% from 2000 [4]. During that same period, the sheep population rose only 10%, and the global cattle population has remained fairly constant at about one billion head [5]. Nearly 60% of the world’s goats are found in Asia, with China, India, Pakistan and Bangladesh having the highest populations. Although most income from global goat production comes from meat sales, there has been a simultaneous increase in goat milk production and consumption.

## GLOBAL DAIRY GOAT PRODUCTION AT A GLANCE

**Figure 1:**
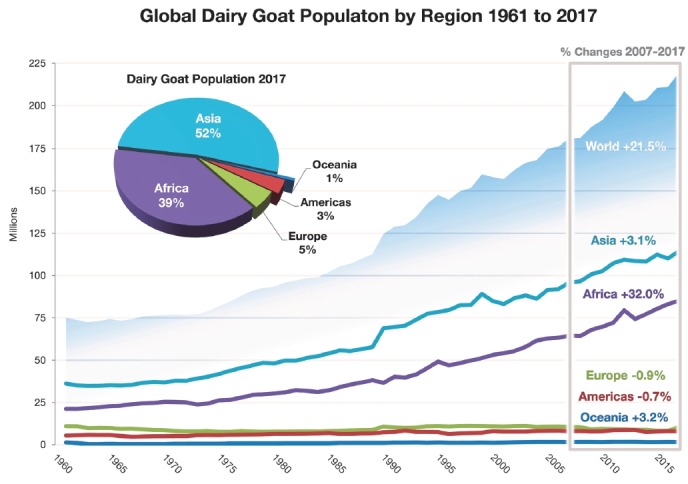
World dairy goat population (heads) during 1961 to 2017 (Compiled from [4], aggregated, may include official, semi-official, estimated or calculated data).

The global dairy goat population was estimated to be 218 million in 2017 [4]. There has been a continuous increase in dairy goat numbers globally, with dramatic increases in the 1990s (Figure 1). In 2017, Asia had the largest proportion of the world population (52%), followed by Africa (39%), Europe (5%), Americas (4%), and Oceania (<1%). During the past decade (2007 to 2017), the world dairy goat population increased by almost 22%. Africa experienced the most rapid increase (32%), followed by Asia (19%), and Oceania (3%), with a minor net decrease observed in Europe (−0.9%) and Americas (−0.7%). Europe contributes 15% of the total goat milk with only 5% of the population, because of greater specialization and commercialization.

**Figure 2:**
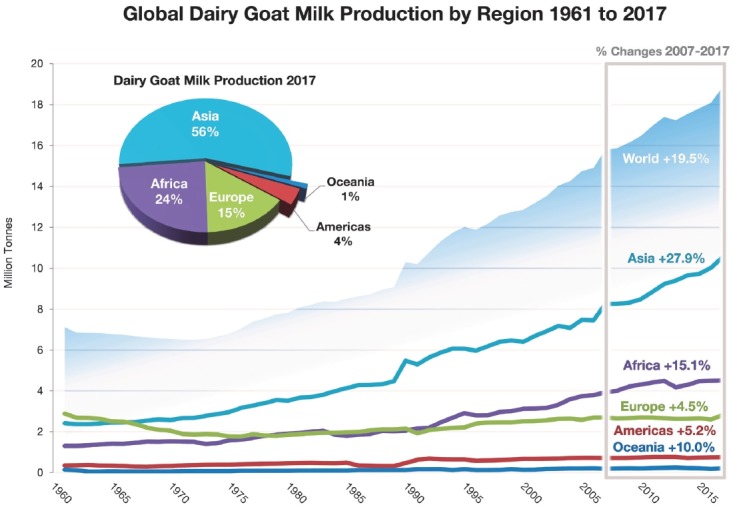
Global trends of goat milk production (Tonnes) from 1961 to 2017 (Compiled from [4], aggregate, may include official, semi-official, estimated or calculated data).

Total global goat milk production was estimated at 18.7 million tonnes in 2017 [4]. It increased 62% from 1993 to 2013 [6]. From just 2007 to 2017, production increased by 16% [4]. The dramatic increase in the 1990s corresponds to the growth of the dairy goat population (Figure 2). During the past decade (2007 to 2017), Asia has seen the largest increase in goat milk production (22%), followed by Africa (13%), and Oceania (9%), Americas (5%), and Europe (4%).

Demand for dairy goat products is rising in both traditional and new markets. Goat milk and products increasingly are preferred for their health and nutritional benefits, including greater digestibility and lipid metabolism, in addition to their taste, compared to cow milk [7].

Goats are found in nearly all countries, and are no longer associated only with low income producers, or dry areas. The attractive price for goat products, especially milk, has brought new producers and investors into the field. High quality goat cheese is still associated with France, but is produced by many countries including Italy, Spain, and the United States. Access to good information, health care, production inputs and technology, improved genetics, transport, and markets remains challenging in many areas, as do consistent and supportive policies and strong producer organizations. The potential for the goat milk industry is quite promising especially for low and medium income countries, but investments are needed to integrate inputs, markets, research, and production infrastructure. Government policy and action are critical to ensure that producers can benefit from rising demand for goat milk.

In many countries, official statistics do not include home consumption or informal market sales where records are not kept, complicating efforts to measure the value of goats. Most of the world’s goat milk is produced and consumed in India, Bangladesh, Pakistan, and Turkey. Goat milk for food and income is also important in the countries of the Mediterranean, the Middle East, Eastern Europe and parts of South America [8]. Most goats are kept by small-scale producers, and are not part of specialized production systems, so it can be hard to estimate the contribution of goat milk to livelihoods. The goats provide milk and meat, but also are important assets in areas without well-developed banking systems. In addition, goats and their products play an important cultural role through gifts and exchanges of live animals, consumption of goat meat during religious celebrations or rituals, and provision of skins, fiber, and fertilizer for crops.

The dairy goat sector is part of the global dairy industry, which includes cow, sheep, buffalo and camel milk and their products [9]. Therefore, the price of goat milk relative to cow milk can influence whether or not a producer will expand or move into other activities. Well-organized cow milk sectors with good infrastructure for transport, processing, and inputs can benefit dairy goat producers as well, as long as consumer demand and supportive policies are in place. In most countries, goat milk is more likely to be consumed locally, whereas cow milk is more likely to enter formal markets for processing. Nevertheless, the goat milk sector is becoming increasingly commercialized, presenting unexpected challenges.

World demand for all dairy products is projected to continue to rise, as consumers become relatively more affluent, and increase their consumption of animal products. Prices to the producer are expected to be 19% higher by 2027 compared to the base period 2015–17 [10]. China is the largest importer of all categories of dairy products, and also imports goat milk powder and whey, especially for the manufacture of baby formula. Most of China’s goat whey is imported from Italy and the Netherlands [11]. The dairy goat industry in China has seen a massive expansion, especially in Shaanxi, Shandong, and Henan provinces, due to governmental recognition of the potential of the sector, and targeted research and financial incentives. Production cannot keep up with demand from factories, however, which raises prices nationally and globally.

Although most of the world goat milk production and consumption is located in Asia, the dairy goat industries from other areas offer some instructive lessons about production and marketing systems, responses to changing consumer demand, and uses of technology. The most organized market for goat milk is found in Europe, especially in France, but also in Spain, Greece, and the Netherlands [12]. Dairy goat production in the United States began growing in the 1980s, and larger commercial farms are becoming increasingly important, driven by rising demand for goat cheese. Dairy goat numbers in the USA doubled from 1997 to 2012 [13].

Genetic selection of dairy goats in Europe and North America has resulted in increased production and longer lactation. The specialized dairy goat breeds used in high income countries therefore have high genetic potential for milk production, and have been exported to many developing countries, through live animal transport, and sales of frozen semen or embryos. These exotic goats have been crossed with local breeds to improve milk production but the results have been mixed [14]. The challenge is to feed and manage crossbred dairy goats to reach their genetic potential. Thus, research on feed sources, especially from crop residues, industrial byproducts, or processed plant matter such as *opuntia* sp. cactus has become especially important for low and medium income countries.

There is increased interest in the genetic value of indigenous breeds of goats, and especially their drought and parasite resistance, now that the changing climate is expanding the planet’s dry areas. Although imported Saanen goats continue to be popular around the world because of their high volume of milk, research to characterize and select for higher production from local dairy goat breeds is increasing. Many desert breeds have milk with higher fat and protein than the Saanen, and this is valued by cheesemakers and processers. There is some urgency to the project because some dairy goat breeds are in danger of disappearing due to indiscriminate crossbreeding [15].

Modern dairy goat production is not limited to confinement operations. In Europe and North America, researchers and producers are revisiting pasture grazing, to reduce costs, maintain natural behaviors, and enhance the environment. Depending on the climate and distance to markets, it may be more economical to graze dairy goats for part or all of the year, rather than purchase or mix a total ration of maize and soybean meal. Goats can utilize browse more effectively than other livestock, and when grazed with cattle, can reduce the parasite burden. Pasture management is a major part of many modern dairy goat operations.

## DAIRY GOATS AND THE ENVIRONMENT

Greenhouse gas emissions (GHG) by livestock contribute to climate change, so strategies to reduce their impact are of increasing importance. The gases are chiefly CO_2_, CH_4_, and N_2_O. The mitigation of enteric CH_4_ emissions in dairy goats is important for economic as well as environmental reasons. Methane produced as a byproduct of microbial fermentation in the rumen represents a net energy loss for dairy goats.

Globally GHG emissions from goats and sheep are about 20% to 25% that of beef cattle and dairy cattle. However, dairy goats are not well studied, and production systems vary so widely that it is difficult to generalize. In terms of unit of milk produced, small ruminants appear to emit more greenhouse gases than large ruminants. Average emission intensity for products from ruminants was estimated at 2.8, 3.4, and 6.5 kg CO_2_ -eq/kg fat and protein corrected milk for cow, buffalo, and small ruminant milk, respectively [16]. Therefore, greenhouse gas abatement and mitigation in dairy goats will be a significant research goal for the future.

Existing GHG mitigation methods for use in dairy goats include use of high-quality forages, protein supplementation of low-quality forages, higher concentrate to forage ratio, and inclusion of fat in the diet [17]. Other mitigation methods include methane (CH_4_) inhibitors such as ionophores, probiotics, acetogens, bacteriocins, archaeal viruses, organic acids, and plant extracts; vaccination against ruminal methanogens; bacteriophages; homoacetogens; hydrogen (H_2_) utilizing acetogenic bacteria; and genetic selection [18]. Naturally occurring secondary compounds in plants such as saponins and tannins may reduce greenhouse gas emission in ruminants, and goats consume and tolerate these compounds well compared to other species. *In vivo* and *in vitro* studies [19,20] revealed that alfalfa saponins reduced protozoa number and inhibited microbial fermentation in the rumen.

Effective manure-management systems in dairy goat production can reduce greenhouse gas emissions. Conventional manure management, covered lagoons, composting, and anaerobic manure digesters have been discussed [18]. Applying manure to soil as soon as possible helps reduce CH_4_ emissions, while storing it for an extended period of time encourages anaerobic decomposition and increases methane output. Additionally, keeping manure dry and avoiding applying it on saturated soil is beneficial. The application of manure shortly before crop or pasture growth can mitigate N_2_O production. Intensive and extensive dairy goat production systems have different GHG profiles. Methane emission is generally lower in dairy goats raised in a confinement system, where they are fed more digestible diets high in concentrate. Dairy goats grazing pasture can produce more methane, because the animals are consuming more fiber and less digestible diets. However, when their manure contribute to plant growth in pastures, more carbon is removed from the atmosphere and sequestered.

Experience with dairy cow waste treatment provides important lessons for dairy goat production. Although goat manure is easier to handle than cow manure, as farms get larger, it must be managed properly to minimize methane production, reduce odors, and to avoid contamination of water resources.

## DAIRY GOATS IN LOW AND MIDDLE INCOME COUNTRIES

Dairy goats can be a key tool to achieve the UN 2030 Agenda for Sustainable Development, adopted in 2016, and its 17 interrelated sustainable development goals (SDGs). The livestock sector in general is shifting from its longstanding focus on production in isolation, to enhancing its contribution to all of the SDGs, including human nutrition, environmental protection and gender equality [21]. The dairy goat subsector will be especially important for achieving these goals, because of the importance of goat milk in the diet of poor children, and women’s strong role in dairy goat ownership and management. Nevertheless, these outcomes will not come about automatically, but require explicit planning and accountability systems [22], to protect and enhance women’s ability to take management and marketing decisions as dairy goats become more profitable.

In low income countries of Africa, Asia, Latin America, and other areas, locally adapted goats are raised for both milk and meat, most of which is sold informally. In pastoralist areas, especially in the drier and drought prone zones, goat milk is highly valued and appreciated, and is often the only protein of the diets of children. In general, government-led research, extension and marketing schemes have prioritized cattle, and overlooked small ruminants in general, and dairy goats in particular, despite their importance to some of the most vulnerable populations. Nonetheless, significant goat milk production is found in many African countries, including Sudan, Mali, Somalia, Kenya and Algeria [23]. In the Mt. Kenya area of Kenya, there are about 200,000 dairy goats, and a thriving market for goat milk, and demand for dairy-type animals. Producers remain poorly organized, however, and there is a great need for technical training and health care [24]. African pastoralists such as the Maasai in Kenya and Tanzania rely on milk from cattle and goats for a significant part of their diet. Increased drought and erratic weather has limited their ability to raise cattle, so a larger portion of their herds are now goats.

In Asia, governments and development agencies identified the dairy goat sector as especially sustainable in the face of climate change, and have invested in many dairy goat projects in the past decade. The Asian-Australasian Dairy Goat Network was first organized in 2012 to link researchers, academicians, policy makers, investors, and dairy goat farmers in Asia-Australasia and beyond [25]. As new and inexperienced farmers enter the dairy goat business due to the premium price for goat milk, there is an urgent need to share information and best practices in the region. Please see the articles on specific countries in this special edition.

South East Asian countries including Indonesia, Malaysia, and Thailand, have developed sustainable systems to integrate dairy goats with plantation production of palm oil, rubber, coffee, and cocoa. The animals graze down weeds between trees, and provide fertilizer through their manure. The trees are more productive and sequester the carbon from the animals, reducing total GHGs [26]. Furthermore, dairy goats can be fed forest byproducts such as palm kernel cake, which when probiotics are added to reduce the lignin, can completely replace concentrate in their ration [27]. Despite the economic and environmental benefits, however, integration of palm oil plantations with livestock remains low. The palm oil production is prioritized, and little training is offered in ruminant production. On large plantations, there may be conflict between the livestock and palm oil managers, and machinery to process the byproducts into feed may be unavailable [28].

In Western Asia and North Africa (WANA or the “Middle East”), the small ruminant population has been increasing since the 1960s, especially in dry areas. Goat keeping is becoming relatively more important as goats adapt better to harsh arid conditions than sheep, and have a higher milk yield [29]. Government support such as animal health services and water points, and assistance during drought with free or subsidized animal feeds, and rescheduling of loans increased goat populations in the region [30], but has not been consistent or of uniform quality.

Where extensive grazing is the main resource for local livelihoods, goats have become an essential aspect of the cultural, social and religious life of the people [31]. There are at least 32 breeds of goats in WANA, adapted to the semi-arid and arid climate as well as to oasis or humid coastal regions. The status of many of the goat breeds in the region is unavailable or undocumented. They are threatened by crossbreeding and small population size [15]. The most significant dairy breed in WANA is the Damascus or Shami goat, originally from Syria but now widely distributed. It has been imported throughout the region to increase milk and meat output through crossbreeding. Although milk and income generally increase through these schemes [32], there are concerns about the loss of genetic diversity.

Scientific knowledge and experience about dairy goats in the Mediterranean basin is generated and exchanged through the Inter-regional FAO/CIHEAM Network for Research and Development in Sheep and Goats. This network, which began in 1970 at the Mediterranean Agronomic Institute of Zaragoza (Spain), is helping to improve production, marketing, environmental management, and communication throughout the Mediterranean and Middle East through research projects, conferences, training and reports [33].

In the Latin American countries, dairy goats make an important contribution to smallholder livelihoods, especially in arid and less favored areas. Mexico, Brazil, and Argentina are middle income countries in the Western hemisphere but have significant low-income populations, especially in the dry areas, and are the main dairy goat-producing countries [34]. Brazil has the most developed dairy goat sector in South America, with strong government investment in research. The Brazilian Agricultural Research system (EMPRAPA) has a National Program of Goat Research (Centro Nacional de Pesquisa de Caprinos do Empresa Brasileira de Pesquisa Agropecuária). The “Fome Zero” (No Hunger) social protection scheme purchases goat milk directly from organized groups of producers in poor and marginal areas in the northeast part of the country, and processes and distributes it to needy families [22].

The International Livestock Research Institute (ILRI) has recognized that goats are relatively more important to the livelihoods of the rural poor, compared to cattle, so investments in goat health, productivity and sales can have greater impact on poverty alleviation. The dairy goat and root crop project in Tanzania showed the great potential and interest in dairy goat interventions to generate income, improve human nutrition, increase gender equality and build community organizations [35]. This comprehensive project included improved goat genetics, training in animal health and production, and gender awareness for both men and women which increased women’s decision-making about productive assets and reduced their workload, leading to improved income and health outcomes for the family. Nonetheless, many government officials in Africa continue to associate goats with “backwardness” and “environmental destruction” so it may be difficult to gain their support for investments in goat production, health and marketing. In the past many dairy goat development projects suffered from a narrow focus on production without including the social context, transport to market, and local preferences. In addition, management of public resources such as rangelands and water sources often need greater input from stakeholders, including the poor and marginalized, to achieve development goals [21].

Development projects with dairy goats can be financially successful if they are well planned and supported. The “Knowledge Harvesting Project on Goats” by IGA/IFAD systematically examined the suitability of goat value chains for lifting people out of poverty and improving food security. Case studies on dairy goat interventions from Kenya, Brazil, and Mexico found a positive internal rate of return in all three countries. In Kenya, where herds are small and goat milk production is not the main livelihood, household net income rose 4 to 6 times, to US$ 600 from US $100–150. In Brazil and Mexico, where the goat enterprise was the main economic activity of the household, net income before labor costs rose to US$ 2,000–11,500 from US$1,000 [22].

## DAIRY GOAT HEALTH

The *Global Peste des Petits Ruminants* (PPR) Eradication Campaign organized by FAO, OIE and individual countries, has brought increased attention to the importance of goats and sheep [36] and has helped to mobilize awareness and resources to improve the goat sector in Africa, Asia and the Middle East. National and international organizations are collaborating to eradicate PPR by 2030, based on the model for the successful eradication of rinderpest in cattle in 2010. PPR has been spreading since 2010, and is now found in most of Africa, the Middle East and much of Asia. It is especially devastating to smallholders who depend on goats and sheep for livelihoods in often precarious circumstances [37].

New research in virology, immunology and genetics is leading to novel types of recombinant vaccines using viral vectors against PPR, as well as Rift Valley fever (RVF), Goat Pox, and Contagious Caprine Pleuropneumonia, which could control the most important production-limiting diseases of small ruminants. Viral antigens are inserted into an adenovirus platform, expressing only one or two proteins, so a vaccinated animal can be distinguished from a naturally infected one. “DIVA” stands for “distinguishing infected from vaccinated animals” and DIVA vaccines are crucial for serological surveillance, especially important for reportable diseases such as PPR and RVF [38].

*Brucella melitesis* is a bacterial disease of goats and sheep, and can infect humans through consumption of raw milk or contact with aborted fetal tissue. The Rev. 1 vaccine has been used to successfully eliminate the disease in high income countries with good animal health services, but can cause abortions in pregnant animals, is virulent for humans, and the antibodies interfere with sero-diagnosis [39]. In low and middle income countries, vaccination has not been used effectively due to cost, and the low priority of goats for policymakers. A new initiative for an improved vaccine is managed by GALVmed [40], and is spurring basic and applied research around the world.

Dairy goats are more likely to transmit zoonotic diseases to humans because many carried through the milk, such as brucellosis. Dairy goats are more likely to be raised in confinement compared to meat goats or fiber-producing goats, which also increases the risk of disease transmission to humans. Reproductive infections caused by *Chlamydophila abortus*, *Coxiella burnetii*, and *Toxoplasma gondii* are causes of infectious abortion in dairy goats. People can be infected by handling fetal membranes or fluids or even inhaling dried Coxiella particles mixed with dust, causing Q fever [41].

High producing dairy goats are at increased risk of mastitis, so farmer training, lab support and access to inputs such as appropriate antibiotics are important. The free online manual “A Guide to Udder Health for Dairy Goats” provides the latest information to assist producers, veterinarians, extension and dairy support personnel in the production of quality goat milk [42].

Delivery of appropriate health, management and marketing information and services to goat keepers in low income countries remains a challenge. Women, pastoralists and the poor are usually not reached by government or private veterinary services, yet they are most likely to depend on goats for food and income security. The International Development Research Center of Canada is funding new research into the most effective entry points and models for delivering vaccines, information and services for women who raise goats [43]. Goat productivity cannot improve without an effective system for quality animal health service delivery that is affordable and accessible.

## LESSONS FROM EUROPE

In Europe, the goat sector is specialized for milk production, which is highly commercialized. The European dairy goat sector is well-regulated, and nearly all goat milk is processed, mostly into cheese. Dairy goat production is more common in the Mediterranean countries of France, Spain, Italy, and Greece, where it is important from an economic, environmental and sociological perspective [33]. The dairy goat sector of the Netherlands has been expanding since 1984 when many dairy farmers transitioned from cows to goats due to quotas for cow milk [44], and the Dutch have become important producers of goat milk in Europe. Because of their familiarity with intensive dairy cow management, Dutch dairy goat producers use more technology such as artificial insemination (AI), confinement rearing, and computerized record-keeping than many other European farmers.

Most fluid goat milk is sold for processing into cheese in factories, or is transformed on the farm, and relatively little is consumed directly by the producer’s family. In France, Spain, and Greece, dairy goats are raised on both family farms and larger commercial intensive operations, often side by side. Years of genetic selection have led to herds with very productive animals. In the Netherlands, the average production per doe is 798 kg/yr; in France, it is 687 kg/yr, and in Spain, it is 352 kg/yr [44]. High productivity is the result of the availability of excellent genetics and widespread use of AI, coupled with high planes of nutrition and good management.

Israel is often grouped with the European countries for statistical purposes because of similar levels of technology and production, as well as trade agreements and sanitary regulations. The average production per doe in Israel is 305 kg/yr, and higher on many specialized goat dairy farms. The average worldwide production is only 90 kg/yr per doe [44].

### The dairy goat sector in France

Historically, goat milk had been part of the human diet, and preferred over cow milk throughout much of Europe, especially in the Mediterranean countries. It was displaced in the 20th century by bovine milk. Cows produce a larger volume of milk, which was easier to collect and transport for industrial processing. By 1950, the industrialization and intensification of agriculture marginalized dairy goat activities in Europe, with legal and sanitary regulations favoring cow milk production and processing, and little investment in scientific research to improve the genetic value of goats or the capacity of their owners [45].

The modern dairy goat industry began in the late twentieth century in central-western France through the initiative of dairy goat cooperatives and cheese enthusiasts [45]. Their success is due to the following (11):

- Demand: The demand for French goat cheese continues to grow both nationally and internationally, both for gourmet and mass market types.
- Government: The French government provides regulations and financial support for production and processing, at both industrial and artisanal levels.
- Quality: Artisanal cheese production (cheese made on the farm) takes place under extremely hygienic conditions, with regular inspections.
- Markets: Most producers belong to marketing networks which help them realize a premium price.
- Image: Consumers associate dairy goat products with responsible natural resource management, good hygiene and strong concern for animal welfare.

Government support has been crucial in the development and continued growth of the French dairy goat industry. Dairy goat producer associations go back as far as 1901 [46], and the professionalization and training of producers, and their inclusion into networks with researchers, input suppliers, and processors has been intentional and well-funded.

The French government supports 3 types of organizations that ensure high quality dairy goat products:

- National professional organizations that provide information to producers and help them exchange experiences and innovation. Some examples are the FNEC (National Goat Farmers Federation), and ANICAP (National Inter-professional Goat Association).
- Technical centers dedicated to research and extension on dairy goats to keep the field responsive to changing consumer tastes. Some examples are the ITPLC (Technical Institute for Dairy Goat Products) and the Goat Centers in Le Pradel, Carmejane, Surgères, and Niort.
- Government support for genetic selection and widespread AI for Alpine and Saanen breeds (Caprigène, Capri AI) have increased productivity per doe, and made AI services available in every part of the country [47].

Other European countries, especially Spain and the Netherlands, have well-developed dairy goat sectors, and they export much of their goat milk to France for processing. Spain also produces “mixed cheeses”, made from mixing the milk of goats and cows, or goats and sheep. In Greece, the goat milk is used to produce traditional cheese (“feta”) mostly for national consumption [48].

Although the goat milk sector is still very significant in Greece, the total goat population has fallen by 24% from 2000 to 2013 reflecting intensification and modernization, with fewer but larger farms replacing many small ones [23]. Also, while traditional pastoral management of dairy goats provided livelihoods for people in dry or mountainous areas, today the dairy goat population is shifting to peri-urban centers where industrial cheese-making plants are located, resulting in significant loss of income in rural areas [49].

### Protected designation of origin and “Terroir”

Goat milk production has remained stable overall in Europe, with slight increases or decreases in some countries. The main market growth has been in the locally rooted “terroir” cheeses with a large number holding a certificate of “Protected Designation of Origin”, or “PDO” [50]. “Terroir” is how a particular region’s climate, soils and topography affect the taste of a fermented agricultural product, such as cheese or wine.

The European Union grants official recognition to regional foods so producers get a premium price for authentic products, and consumers can make informed purchases. The system began in 2012, and is parallel to the national naming or “appellation” systems in the member countries, such as *appellation d’origine contrôlée* (AOC) used in France which restricts the use of certain names for cheeses based on their geographic origin. The PDO is only granted to foods produced in a geographic area, and made in a specified manner. The label requires very strict documentation and monitoring, but leads to a premium price for the product.

The PDO label is especially important to the European goat cheese sector compared to cheese made from cow milk, despite the vastly larger volume of the latter. Out of 164 cheese types with a PDO or a protected geographical indication listed on the website of the European Commission, nearly half are made at least in part with small ruminant milk [51].

The integration of markets throughout the European Union has helped to transport milk to French factories for processing and to distribute finished products throughout Europe and beyond. The steady reduction of governmental intervention in the EU dairy market, however, has resulted in greater market orientation on the one hand, but also diminishing income security for farmers on the other. Producers using high levels of purchased inputs are especially vulnerable to market price fluctuations. Most high producing dairy goats in Europe consume a ration that includes soybeans and maize, in addition to hay or pasture, depending on the season. When soy and maize prices rise, and the price of milk does not, farmers can lose their business. During the grain price shocks of 2011, Spain lost 30% of its herd when owners had to sell their animals [52].

### Consumption of goat dairy products in Europe

Goat milk is part of the historical “Mediterranean Diet”, famous for its health benefits and recognized as part of the Intangible Cultural Heritage of Humanity by the United Nations [53]. In Europe today, there is a renewed appreciation for the taste and health benefits of goat milk, and pride in the traditional diet, leading to increased consumption. Also, European consumers associate goat milk with natural, rural and sustainable farming, and therefore choose to purchase it to support a way of life they value [47]. The welfare of farm animals is among the top three issues that European consumers want to know more about, after safety and quality of foods, and the effect of agriculture on environmental and climate change [54]. Producers and government regulations take care to meet their consumers’ expectations for environmental management and animal welfare. There is increasing information available on natural goat behaviors, and how to meet them in modern goat production systems, [55] but more research and farmer training in needed.

### Social and environmental impacts

The dairy goat sector is used to accomplish social and environmental goals at the national and regional level in many countries in Europe. For example, goats have a recognized role in maintaining biodiversity, landscape conservation for tourism, and in land management to combat forest fires. Beginning in the 20th century, traditional pastoralism or movement of goat herds to take advantage of changing natural pastures, was discouraged. Often pastoral communities were situated in remote, dry, mountainous or less desirable land, and became impoverished when livestock movements were restricted, or national priorities marginalized them. To combat rural poverty, many governments now support grazing and pastoral management through financial incentives, recognizing the positive environmental impact of controlling weeds and shrubs and preventing fires [50]. In Spain, a tool called RAPCA, the “Grazed Fuel Break Network in Andalusia” intentionally promotes pastoral movement of goats to prevent fires [56]. Investments in technical training and marketing enable pastoral communities to preserve their heritage while improving livelihoods.

Another priority of many European Ministries of Agriculture is to encourage “agro-ecological” or “organic” livestock production practices. The French Ministry of Agriculture is implementing a program of training and incentives to reduce livestock producers’ dependence on purchased feed through increased use of pasture and browse. They also promote improved management so that less medication or chemical inputs are used. This is especially beneficial for goat producers because goats can take advantage of pasture and browse more effectively than other ruminant species, especially in hilly, mountainous and dry ecosystems [57]. Reduced use of antibiotics in livestock is essential for preventing antimicrobial resistant bacteria, which threatens control of infectious disease in both humans and animals throughout the world [58].

Agro-ecological practices can allow dairy goat producers to label their cheeses as “organic”, which is increasingly attractive to consumers concerned about the impact of high levels of fertilizers, synthetic pesticides, antibiotics, hormones and fossil fuels on water and air quality, as well as human health. Agro-ecological dairy goat farming can increase farm incomes but requires skilled management and strong investments in research, extension and marketing.

## THE AMERICAS

The history of dairy goat production is quite different in the Americas because goats are not indigenous to the Western Hemisphere. European breeds were introduced during the colonial period, beginning in the 1590s by the Spanish, and are still the most popular dairy breeds. Nubian goats from Egypt via England, and Nigerian Dwarf goats from West Africa have been introduced as well, but the Swiss and other European breeds remain dominant. Most commercial dairy goat herds include crosses or grade animals.

As in Europe, the dairy cow industry in the United States and Canada is well developed, and agricultural research and extension is excellent. Supply chains for machinery, feeds, medicine, and vaccines are well-established. The dairy regulatory framework is clear and enforced, and infrastructure and transportation are available. But goats are not little cows, and the cow model cannot be applied to the dairy goat industry without modifications. A more detailed discussion on dairy goat production in Americas has been presented separately [59].

### Lessons from the United States

The dairy goat sector in the USA is still quite small by global standards, and when compared to the dairy cow industry, but is growing quickly. Out of the estimated 2.6 million goats in the USA in 2018, 380,000 (16%) are thought to be raised primarily as dairy animals [60], a 12% increase from 2012, and dairy goat numbers doubled from 1997 to 2012 [13].

Information on and for dairy goats in the USA is relatively sparse because goats have been considered a “minor use” species, creating serious limitations for producers, manufacturers, and policy makers. For example, only 15 drugs are approved for use in goats, and most cannot be used in lactating does [61]. Statistics on goats are relatively new to the National Agricultural Statistics Service (NASS), and the first goat survey was conducted in 2005. Goat production is becoming more commercialized, but markets are still highly informal, and goats may be kept for multiple purposes, which limits the quality of information collected through traditional surveys [62].

Rising demand is driving the increase in dairy goat production. Changing consumer demographics is a major force, because populations with Mexican, African, and Middle Eastern heritage often prefer goat to cow milk. Affluent urban consumers appreciate the taste of French style goat cheeses, and many believe that goat milk products confer more health benefits than cow milk. Goat cheeses produced in the USA are now very high quality, and have won numerous awards at international competitions [63]. The “locovore” movement encourages consumers to purchase and consume foods from local, small-scale producers to build social stability, and enhance environmental stewardship by decreasing transport costs. There are many small-scale goat farms surrounding the major cities on the East Coast of the United States, supplying the growing numbers of “locovore” consumers with cheese, fresh milk and yogurt.

### The legal and regulatory environment for selling goat milk in the US

The regulations for the dairy industry were developed for cows, creating a difficult barrier when goat milk producers began applying for licenses in the 1980s. In 2006, after many scientific studies and years of lobbying, the Dairy Practices Council published the “Guidelines for the Production and Regulation of Quality Dairy Goat Milk” which allowed the sector to flourish within a legal framework [64].

There are significant differences in the national standards for cow and goat milk. In 1991, the minimum somatic cell count (SCC) for “Grade A” (highest quality) cow milk was set at 750,000 cells/mL, but milk from healthy goats will test much higher. Therefore, in the 2006 Guidelines, goat milk was permitted to have an SCC of 1 million cells/mL. This was raised to 1.5 million somatic cells/mL for goat milk in 2009, making it possible for more producers to market “Grade A” goat milk, which commands a premium price. The national Food and Drug Agency standardized fluid goat milk to contain a minimum of 3.25 percent fat and 8.25 percent milk solids not fat (or the sum of the protein, lactose, and minerals). Ensuring the uniformity and legality of finished dairy goat products expands the market, and is good for both producers and consumers [65].

### Helping producers become more professional

Recording and using data to improve dairy goat production distinguishes professional or modern goat keeping from traditional or low input strategies, where simple survival of the animals may be the main objective. In the United States, official testing and record keeping is done by the Dairy Herd Improvement Association (DHIA), a combined national and state program of milk testing and record keeping that charges a fee for monthly farm visits. The weight of milk produced by each goat is recorded, along with other data such as milk fat, and reproductive performance. Recommendations can be made, and genetic merit can be established using herd health analytical software.

The cost can be prohibitive for small-scale dairy goat producers, because there are no subsidies available. Dairy cow businesses can absorb the cost more easily because of their greater sales [7]. Still, use of DHIA is increasing, from less than 1% of dairy goat herds in 2004 to 13% in 2012. In France, where government and producer groups subsidize the cost of testing, 95% of herds participate, which represent 85% of their dairy goats, resulting in improved productivity [66].

The US dairy goat industry is where the cow sector was about 15 years ago, regarding use of machinery for milking and feeding animals, computerized management, AI, marketing and specialized support from nutritionists, extension agents and veterinarians [13]. Training programs are essential for producers to become more professional, to take advantage of new technology, and to access bank loans. . Many universities now have extensive online resources for dairy goat production and marketing, including certification programs. Most are free, and available to users in all countries.

Langston University, American Institute for Goat Research: http://www.luresext.edu/

Michigan State University Sheep and Goat Extension https://www.canr.msu.edu/sheep_goats/

Cornell University Goat Program: https://blogs.cornell.edu/goats/

University of Maryland: https://extension.umd.edu/cecil-county/4-h-youth/dairy-goat

Penn State Extension: https://extension.psu.edu/dairy-goat-production

Iowa State University: https://www.extension.iastate.edu/dairyteam/dairy-goats-and-sheep

North Carolina State University Extension: https://meatgoats.ces.ncsu.edu/2017/06/new-goat-management-book-for-dairy-goat-farmers/

There is also an excellent set of online resources from Canada:

Onterio, Canada: https://ontariogoat.ca/producer-info/resources/

All 50 US states have at least one goat breeders’ association, and there are many national organizations, local clubs, websites, magazines, shows and fairs to help producers learn from each other, and reach consumers. Innovative products such as new goat cheeses, candy and cosmetics made from goat milk introduce new customers to the industry [67]. Producer groups help to market dairy goat products, but their impact is limited because they are small and entirely self-funded. There is little government support for the dairy goat industry in the US except for policies like sanitary regulations.

In 2019, the trade in dairy goat products is global. There is increasing consolidation of goat milk manufacturing, although most US produced cheese is not exported but consumed within country. Still, dairy goat production is increasingly profitable, attracting the attention of global dairy processors trying to participate in the market [59].

## CONCLUSION

The European model shows that the dairy goat sector can be modern, sanitary, and profitable, with high quality products and global markets. The US example shows how a new dairy goat industry can evolve quickly when interested producers and consumers reach a critical threshold, and the regulatory framework designed for cows changes to accommodate goat milk. In all cases, government and producers must work together to protect public health, and farm gate prices must incentivize clean milk. Fair prices from processors should be based on fat and protein rather than volume, which helps producers improve their management decisions.

There is increasing pressure on smallholder dairy goat producers to commercialize and intensify their operations, or lose their livelihoods. Training on health, nutrition, reproduction and management are essential but require strong and inclusive organizations, which may be difficult for smallholders to form without outside assistance. Other investments, however, will be ineffective without such producer organizations. The professionalization of dairy goat producers in France was supported by government agricultural policy that included facilitation of strong and trustworthy producers’ unions.

National policies must be developed with the participation of all actors in the field because they determine who will benefit from the growing market. Large and small-scale producers can coexist to meet market demand, provide employment, and manage the natural resource base in a sustainable manner. In low and middle income countries, investments in extension and training, milk testing and sanitation, and reliable transportation and handling of raw milk and milk products are needed. Because dairy products are extremely perishable, protection of public health is a priority, but this should not be a reason to exclude smaller producers. Small-scale farmers can produce a nutritious and sanitary product with the right support, pricing and incentives, leading to further social as well as economic benefits. As with any other livestock, positive environmental impacts do not happen by chance, but are the result of evidence-based regulations, adequate financial support, community involvement, sensible incentives, clearly defined outcomes, and a clientele willing to pay a fair market price for quality products. As the global dairy goat industry continues to integrate, the dichotomy between subsistence and commercial production persists, and both systems may co-exist within the same country. The challenge is to facilitate subsistence producers’ transition to commercialization by understanding and addressing their motivations and obstacles.

Important areas for future research on dairy goats includes greenhouse gas mitigation, cost effective rations made from unconventional feedstuffs and byproducts, novel vaccines that are thermotolerant, and permit infected animals to be differentiated from vaccinated ones (DIVA), and genetic progress through molecular analysis. New models to integrate diverse producers with service providers, markets and policy makers will be necessary to manage coming changes. Finally, consumers are demanding greater attention to animal welfare, so our research and extension systems must respond.

The growing global market for dairy goat products can be good for producers and consumers of all countries. Competition can lead to innovation, improved quality and greater choice for consumers. Scientific research on dairy goats is no longer a marginalized topic, but is increasing globally and becoming more mainstream [67]. Scientific cooperation and information exchange have begun in dairy goat nutrition, reproductive technology, and genetics, but we need more opportunities to bring researchers, producers, policy makers and development workers together to share best practices for farmer support strategies, environmental management, and consumer education. Together, we can improve the quality and popularity of dairy goats and their products, and keep the market healthy for the future.
